# Nuclear expression of Lyn, a Src family kinase member, is associated with poor prognosis in renal cancer patients

**DOI:** 10.1186/s12885-016-2254-9

**Published:** 2016-03-16

**Authors:** Antonia K. Roseweir, Tahir Qayyum, Zhi Lim, Rachel Hammond, Alasdair I. MacDonald, Sioban Fraser, Grenville M. Oades, Michael Aitchison, Robert J. Jones, Joanne Edwards

**Affiliations:** Wolfson Wohl Cancer Research Centre, Institute of Cancer Sciences, College of Medical, Veterinary and Life Sciences, University of Glasgow, G61 1QH Glasgow, Scotland UK; NHS Greater Clyde and Glasgow, Southern General Hospital, G51 4TF Glasgow, Scotland UK; NHS Greater Clyde and Glasgow, Gartnavel Hospital, G12 0YN Glasgow, Scotland UK; Beatson West of Scotland Cancer Centre, Institute of Cancer Sciences, College of Medical, Veterinary and Life Sciences, University of Glasgow, G12 0YN Glasgow, Scotland UK

**Keywords:** Renal cell carcinoma, Src family kinases, Paxillin, Dasatinib, Apoptosis, Phosphorylation, Wound healing, Human cell lines

## Abstract

**Background:**

8000 cases of renal cancer are diagnosed each year in the UK, with a five-year survival rate of 50 %. Treatment options are limited; a potential therapeutic target is the Src family kinases (SFKs). SFKs have roles in multiple oncogenic processes and promote metastases in solid tumours. The aim of this study was to investigate SFKs as potential therapeutic targets for clear cell renal cell carcinoma (ccRCC).

**Methods:**

SFKs expression was assessed in a tissue microarray consisting of 192 ccRCC patients with full clinical follow-up. SFK inhibitors, dasatinib and saracatinib, were assessed in early ccRCC cell lines, 786-O and 769-P and a metastatic ccRCC cell line, ACHN (± Src) for effects on protein expression, apoptosis, proliferation and wound healing.

**Results:**

High nuclear expression of Lyn and the downstream marker of activation, paxillin, were associated with decreased patient survival. Conversely, high cytoplasmic expression of other SFK members and downstream marker of activation, focal adhesion kinase (FAK) were associated with increased patient survival. Treatment of non-metastatic 786-O and 769-P cells with dasatinib, dose dependently reduced SFK activation, shown via SFK (Y^419^) and FAK (Y^861^) phosphorylation, with no effect in metastatic ACHN cells. Dasatinib also increased apoptosis, while decreasing proliferation and migration in 786-O and 769-P cell lines, both in the presence and absence of Src protein.

**Conclusions:**

Our data suggests that nuclear Lyn is a potential therapeutic target for ccRCC and dasatinib affects cellular functions associated with cancer progression via a Src kinase independent mechanism.

## Background

In the UK, 8000 new cases of clear cell renal cancer (ccRCC) are diagnosed each year; survival rates are poor, with estimated five-year survival rates at only 50 %. Currently the mainstay drug therapy for advanced renal cancer is vascular endothelial growth factor receptor tyrosine kinase inhibitors (VEGFR TKi, such as sunitinib, pazopanib and axitinib) and inhibitors of mammalian target of rapamycin (mTOR, such as everolimus or temsirolimus) [1–3]. Despite these recent advances, the outlook for these patients remains poor with little prospect of a cure. Further insights into activated signalling pathways in renal cancer will help guide development of improved therapies.

The non-receptor tyrosine kinase Src is associated with multiple oncogenic cellular processes including migration, adhesion, invasion, proliferation and survival [4]. Src kinase is the prototypical member of the Src family kinases (SFKs), with a total of 8 members expressed in mammalian cells (Src, Blk, Fgr, Fyn Yes, Hck, Lck & Lyn). Autophosphorylation of the Y^419^ site in the kinase domain of Src can be used as a marker of activation; however due to high levels of similarity in this region between SFKs, the Y^419^ site is not specific to Src, but is associated with activation of all family members [5, 6]. Therefore, autophosphorylation of Y^419^ cannot be assumed to represent only Src kinase activation, instead all SFKs need to be investigated to dissect out which member is being affected in different tumours.

SFKs are elevated in various malignancies including prostate, breast, colon and lung [7–10]; however depending on the tumour type they can be associated with good or poor prognosis [8]. To date there is limited evidence examining SFK expression in renal cancer. Suwaki et al. (2011) reported that in metastatic renal cancer the HIF-regulated VHL-PTP1B-Src kinase signalling pathway determines the sensitivity of tumours to the SFK inhibitor, dasatinib [11]. Previous work in our laboratory observed that SFKs are expressed at their highest level in T2 stage carcinomas, suggesting that the SFK pathway is active in ccRCC prior to development of metastasis [12]. Accordingly, there is a need to establish which SFKs are associated with poor prognosis in ccRCC and to determine the mechanisms of action of SFK inhibitors in early (non-metastatic) and late (metastatic) stage tumours.

The aims of the current study were to evaluate whether SFKs and downstream markers of activation were associated with ccRCC patient prognosis and to investigate the functional effects of two SFK inhibitors, dasatinib and saracatinib, in non-metastatic and metastatic ccRCC cell lines.

## Methods

### Materials

Dasatinib and saracatinib were obtained from Selleck Chemicals (Texas, USA). Src ONTarget Plus smartpool small interfering ribonucleic acid (siRNA) and non-targeting (NT) ONTarget Plus siRNA #1 were acquired from Thermo Fisher Scientific Biosciences GMBH (Loughborough, UK). All other reagents were obtained from Sigma, UK unless otherwise stated in the text.

### Patient cohort

The tissue microarray (TMA) consisted of 192 patients diagnosed with ccRCC within the Greater Glasgow NHS Trust between 1997 and 2008; all patients gave informed consent prior to biopsy for samples to be used for research purposes. These patients had undergone complete resection of the tumour at the time of nephrectomy. Patients were staged pathologically and graded according to the TNM classification and Fuhrman grading respectively. The research ethics committee of West of Scotland approved the study (GN10SU229).

### Immunohistochemistry

Antibodies were validated by a single band on a western blot and with antibody blocking experiments on renal tissue sections. TMA sections were de-waxed and rehydrated through graded alcohols then antigen retrieval was performed by heating TMA sections under pressure for 5 min using either citrate buffer pH6 (Src kinase, focal adhesion kinase (FAK, Y^861^), FAK (Y^397^), Fyn, Hck, Lck, Yes) or Ethylenediaminetetraacetic acid (EDTA) buffer pH9 (Src (Y^419^), paxillin, Lyn, Fgr). TMA sections were blocked with 3 % hydrogen peroxide followed by 5 % normal horse serum (Vector Laboratories, USA) in antibody diluent (DAKO, Denmark) for 20 min at room temperature. Incubation with primary antibody for 60 min at room temperature for Src kinase (1:200) and Fgr (1:4000, Cell Signaling Technologies. USA) or overnight at 4 °C for Src (Y^419^; 1:25, Millipore, UK), FAK (Y^861^; 1:200, Invitrogen, UK), FAK (Y^397^; 1:200, Abcam, UK), Fyn (1:1500), Lyn (1:25), Hck (1:1000), Lck (1:200), Yes (1:150) and paxillin (1:25, Cell Signaling Technologies, USA). Protein expression was amplified and visualized using DAKO envision kit (DAKO, USA) and Chromogen 3,3’-diaminobenzidine (DAB, Vector Laboratories, USA). Sections were counterstained with haematoxylin, dehydrated and mounted. Protein expression was individually assessed at the membrane, cytoplasm and nucleus for each core (three per tumour specimen) using the weighted histoscore method by two independent observers and agreement between observers was calculated using the interclass correlation co-efficient [13]. The average score for each cellular location within individual tumours was calculated and divided into high expression (above median) or low expression (below median).

### Cell culture

786-O (ATCC®, CRL-1932™) and 769-P (ATCC®, CRL-1933™) early non-metastatic ccRCC cells were maintained in RPMI-1640 medium supplemented with 10 % foetal calf serum, 1 % Glutamax and 1 % Penicillin (10,000 Units/ml)/Streptomycin (10,000 Units/ml). ACHN metastatic ccRCC cells (ATCC®, CRL-1611™) were maintained in Essential Modified Eagle's Medium (EMEM) supplemented with 10 % Foetal calf serum, 1 % Glutamax and 1 % Penicillin (10,000 Units/ml)/Streptomycin (10,000 Units/ml). Cells were authenticated using short tandem repeat DNA profiling. Prior to treatment or siRNA transfection, cells were seeded at a density of 1×10^5^ cells/well for 6-well plates and 5×10^3^ cells/well for 96-well plates then incubated at 37 °C with 5 % CO_2_ in air for 24 h. Inhibitor treatments were then performed as appropriate; dasatinib was added for 48 h and saracatinib for 24 h at 37 °C with 5 % CO_2_ in air.

### siRNA transfection

200 nM NT control or Src siRNA was transfected into cells using lipofectamine siRNAMax in optimem-1 as per manufacturer’s instructions and cells incubated at 37 °C with 5 % CO_2_ in air for 72 h. After 8 h incubation, the media was changed to RPMI-1640 or EMEM media and inhibitor treatments performed as appropriate.

### Preparation of cellular extract and immunoblotting

Cell lysates and electrophoresis was performed as previously described [7]. Immunoblotting of Src (Y^419^; Millipore, USA) [14], Src kinase (Cell Signaling Technology, USA) [15], FAK (Y^861^; Invitrogen, UK) [16] and FAK (Y^397^; Abcam, UK) [17] were performed at 1:1000 dilutions with rabbit anti-human antibodies. Proteins were visualized by addition of a 1:5000 dilution of horseradish peroxidase (HRP)-conjugated polyclonal anti-rabbit IgG. HRP-conjugated antibodies raised against beta tubulin were used as loading controls. HRP-conjugated protein was visualized using an enzyme-linked chemiluminescence reaction (Thermo Fisher Scientific, UK) and quantified using a LAS3000 image analyser (Fujifilm, Tokyo) and analysed with Image J software (NIH, US).

### WST-1 proliferation assay

Cell viability was measured by mitochondrial dehydrogenase induced cleavage of water soluble tetrazolium salt (WST), via incubation with WST-1 for 2 h at 37 °C with 5 % CO_2_ in air (Roche Applied Science, UK). Absorbance was then measured at 450 nm.

### Cell death detection ELISA Plus

Cell Death Detection ELISA Plus kits (Roche Applied Science, UK) were used to measure apoptosis by quantifying histone-DNA complexes generated after inhibitor treatment, as per the manufacturer’s protocol. HRP cleavage of 2,2-azinobis(3-ethylbenzothiazoline-6-sulphonic acid, ABTS) substrate was measured by absorbance at 405 nm.

### Wound healing assay

Cell monolayers had scratches created with a pipette tip and were washed twice with PBS. Cells were then treated with inhibitors and incubated at 37 °C with 5 % CO_2_ in air for 20 h. Cell scratches were photographed at 0 and 20 h and the width of the scratch recorded using an Olympus IX51 microscope. The distance refilled between the furthest migrated cell and the scraped edge on both sides was used to evaluate migration.

### Statistical analysis

For immunohistochemistry, disease specific survival (DSS) curves, which represents the time to a cancer-specific death from diagnosis, was generated using the Kaplan-Meier method. The log rank test was utilized to compare significant differences between subset groups using univariate analysis. Multivariate Cox regression analysis was performed to identify those factors that were independently associated with disease specific survival.

All other assays were analysed using a two-way ANOVA followed by Bonferroni post-hoc tests. Statistical significance was set at *p* < 0.05 and experiments were repeated three times.

## Results

### Localisation-dependant associations between disease specific survival and SFK pathway

The relationship between clinicopathological characteristics of patients with ccRCC and disease specific survival is shown in Table 1. In agreement with previous work, SFKs were expressed at highest levels in T2 patient tumours (data not shown).

**Table 1:** Clinicopathological characteristics for ccRCC patient cohort and disease specific survival (*n* = 192)

		Univariate survival analysis^b^		Multivariate survival analysis^c^	
		Disease specific survival		Disease specific survival	
Clinico-pathological characterisitcs	Patient (*n* (%))	Hazard ratio (95 % CI^a^)	*p*-value	Hazard ratio (95 % CI^a^)	*p*-value
Age (≤61/>61)	94 (49)/98 (51)	0.97 (0.50–1.86)	0.916		
T-stage (I/II/III/IV)	83 (43)/33 (17)/69 (36)/7 (4)	2.13 (1.46–3.12)	**9.4×10** ^**−5**^		
Grade (I/II/III/IV)	15 (8)/59 (30)/84 (44)/34 (18)	2.16 (1.40–3.35)	**0.001**	1.56 (1.02–2.40)	**0.042**
Recurrence (None/recurred)	142 (74)/50 (26)	11.47 (5.22–25.20)	**6.78×10** ^**−15**^	12.50 (5.09–30.64)	**1.0×10** ^**−7**^
KLINTRUP (Low/High)	96 (50)/96 (50)	0.97 (0.50–1.87)	0.932		

^a^CI = Confidence Interval

^b^Univariate analysis was performed using the Log Rank Test

^c^Multivariate analysis was performed using Cox Regression

*P*-values in bold are greater than the significance threshold of 0.05

**Fig. 1:**
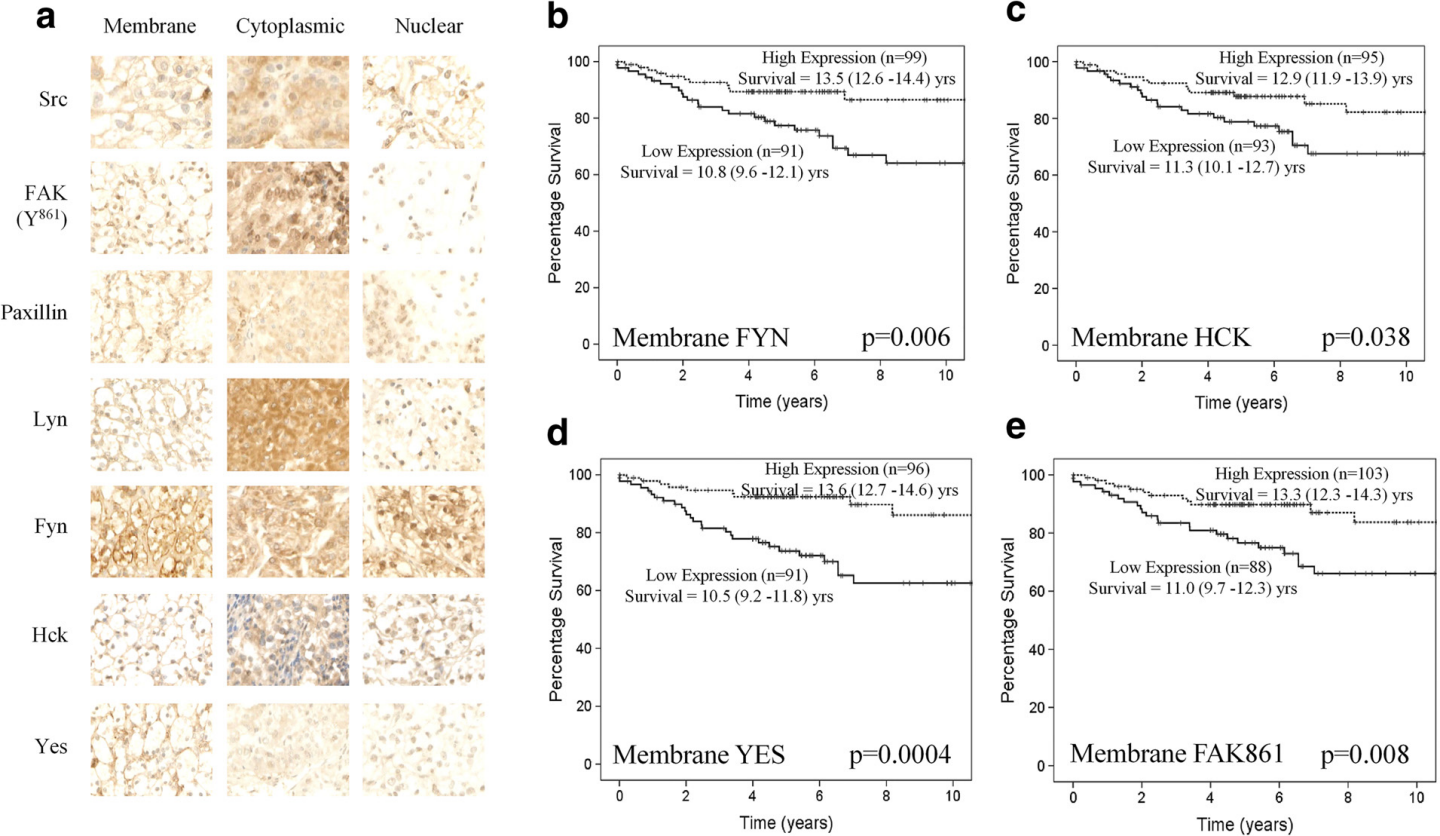
High membrane expression of SFK pathway members increases disease specific survival in a ccRCC patient cohort. **a** Representative pictures showing membrane, cytoplasmic and nuclear staining for SFK family members and downstream targets. (**b**–**d**) Kaplan Meier curves showing high membrane expression of (**b**) Fyn and (**c**) Hck and (**d**) Yes significantly increased disease specific survival by 2–3 years in a cohort of 192 ccRCC patients. **e** Kaplan Meier showing high levels of FAK phosphorylation at Y^861^ within the cellular membrane also increases disease specific survival by 2–3 years. Data shown is the average histoscore for 3 cores per patient and significance was set at 0.05

Expression was independently assessed in cell membranes, cytoplasm and nuclei for each protein investigated (Fig. 1a). Patients whose tumours expressed high levels of membrane Fyn (*p* = 0.006), Hck (*p* = 0.038) and Yes (0.0004) were observed to have a significantly longer disease specific survival compared to those patients whose tumours expressed low levels of these SFK (Fig. 1b-d). In addition, high levels of phosphorylated FAK (Y^861^, *p* = 0.008), a downstream marker of activation, were also associated with good prognosis (Fig. 1e). No significant associations were observed for membrane expression and any other protein investigated (Table 2). When membrane expression of these significant findings was entered into multivariate cox regression model with tumour stage, tumour grade and recurrence, high membrane Yes expression (*p* = 0.014) was demonstrated to be independent of other factors in the model. No significant observations were made between survival and cytoplasmic expression of any proteins investigated (Table 2).

**Table 2:** Protein expression for SFKs and downstream markers of activation and disease specific survival (*n* = 192)

Protein	Median (IQR^a^)	*p*-value
Src Kinase		
Membrane	50 (27–80)	0.237
Cytoplasmic	37 (27–68)	0.132
Nucleus	2 (0–12)	0.726
SFK (Y416)		
Membrane	5 (0–13)	0.206
Cytoplasmic	7 (0–20)	0.868
Nucleus	2 (0–11)	0.735
FAK (Y861)		
Membrane	13 (3–37)	**0.008**
Cytoplasmic	7 (0–20)	0.546
Nuclear	60 (43–77)	0.532
FAK (Y397)		
Membrane	93 (41–147)	**0.001**
Cytoplasmic	77 (33–133)	0.252
Nuclear	152 (127–180)	0.846
Paxillin		
Membrane	3 (0–10)	**0.050**
Cytoplasmic	7 (0–10)	0.335
Nuclear	3 (0–63)	**0.031**
FYN		
Membrane	15 (2–30)	**0.006**
Cytoplasmic	8 (2–17)	0.393
Nuclear	13 (0–109)	0.139
LYN		
Membrane	37 (10–77)	0.141
Cytoplasmic	70 (38–202)	0.446
Nuclear	13 (0–109)	**0.019**
FGR		
Membrane	7 (0–22)	0.087
Cytoplasmic	33 (15–60)	0.252
Nuclear	9 (3–27)	0.503
HCK		
Membrane	63 (27–103)	**0.038**
Cytoplasmic	47 (30–27)	0.109
Nuclear	57 (30–80)	0.197
YES		
Membrane	47 (17–70)	**0.0004**
Cytoplasm	38 (23–69)	0.271
Nucleus	43 (27–62)	0.842

^a^IQR = interquartile range

*P*-values in bold are greater than the significance threshold of 0.05

**Fig. 2:**
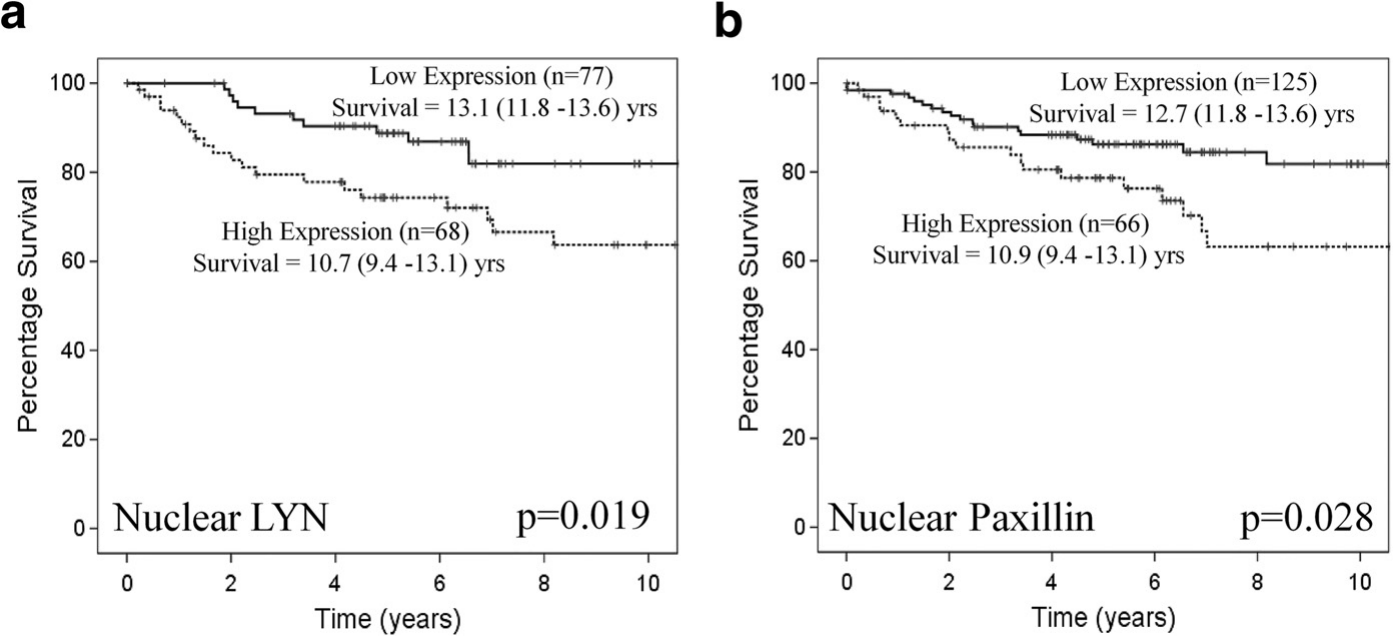
High nuclear Lyn and paxillin expression decreases disease specific survival for patients with ccRCC. Kaplan Meier curves showing that high nuclear expression of (**a**) Lyn and (**b**) Paxillin significantly decreased disease specific survival by 2–3 years in a cohort of 192 ccRCC patients. Data shown is the average histoscore for 3 cores per patient and significance was set at 0.05

In contrast to the association with good prognosis observed in the membrane, patients with tumours that expressed high levels of nuclear Lyn or Paxillin (downstream marker of activation) had significantly shorter disease specific survival compared to patient tumours expressing low levels (*p* = 0.019 & *p* = 0.028, Fig. 2a-b). When nuclear expression of Lyn and Paxillin were entered into multivariate cox regression model with tumour stage, tumour grade and recurrence high membrane Lyn expression (*p* = 0.011) was demonstrated to be independent of other factors in the model.

### Dasatinib and saracatinib affect the SFK pathway in early ccRCC cell lines

**Fig. 3:**
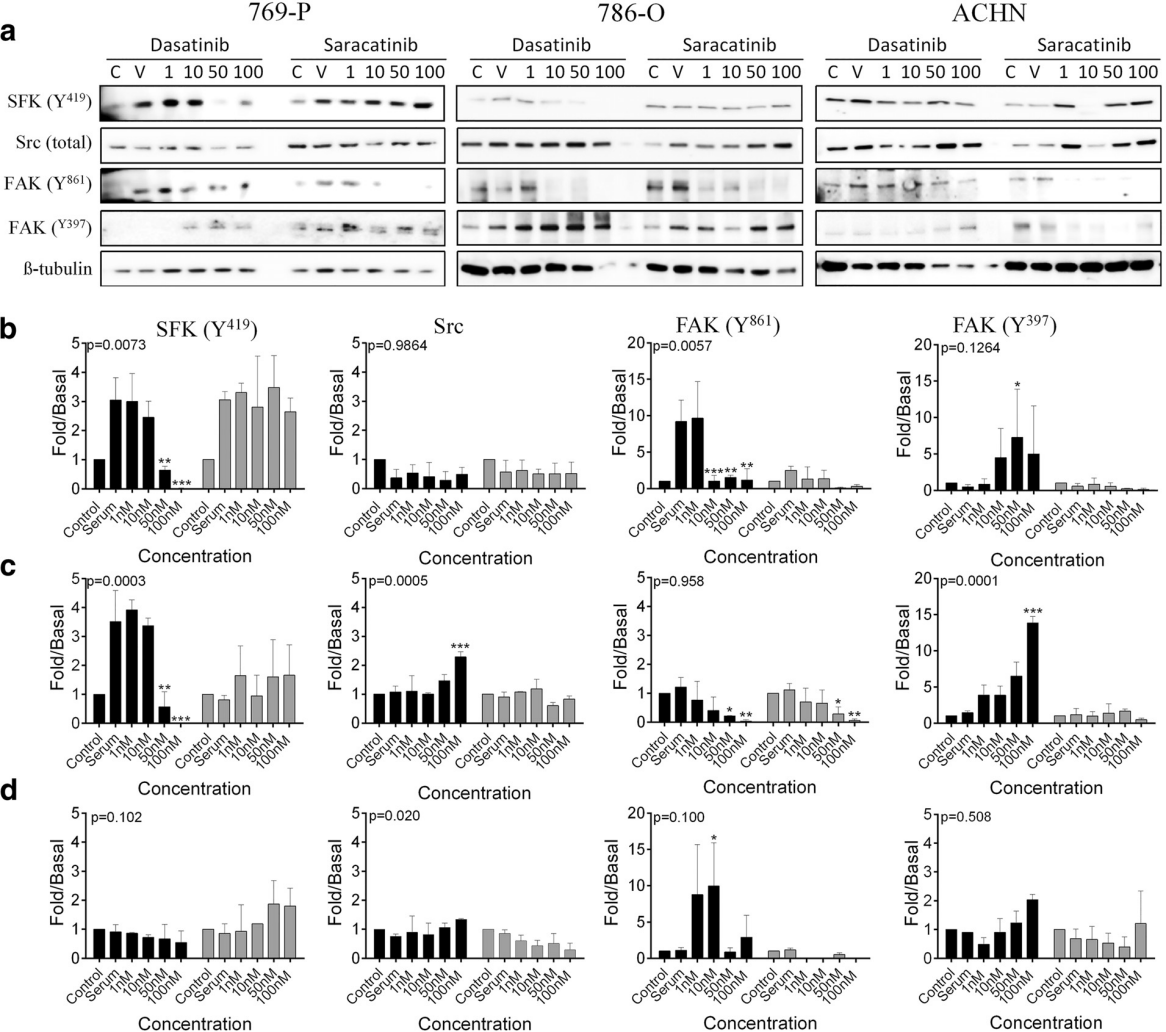
SFK inhibitors affect phosphorylation in non-metastatic ccRCC cell lines. **a** Western blots showing the effect of control (C), 10 % Serum (V), 1, 10, 50 or 100 nM dasatinib or saracatinib on SFK (Y^416^), FAK (Y^861^) and Src kinase in the non-metastatic, 769-P and 786-O and metastatic ACHN cells. **b**–**d** Quantification of western blot results for dasatinib (black bars) and saracatinib (grey bars) in (**b**) 769-P, (**c**) 786-O and (**d**) ACHN cells (*n* = 3-4: * = 0.05, ** = 0.01, *** = 0.001)

In the early non-metastatic, 769-P and 786-O ccRCC cell lines, dasatinib significantly inhibited SFK phosphorylation at Y^419^, however saracatinib has no effect. Neither inhibitor affected the total amount of Src kinase in the cell. However, dasatinib and saracatinib did inhibit phosphorylation of the downstream marker of activation FAK at the Src-dependent site Y^861^, although only dasatinib reached statistical significance (Fig. 3a–c). Dasatinib also caused a significant dose dependent increase in phosphorylation of FAK at the auto-phosphorylation site Y^397^ in non-metastatic 769-P and 786-O cells (Fig. 3a–c). No effect of either inhibitor was noted in the metastatic ACHN cells (Fig. 3a, d).

### SFK inhibitors affect cellular functions associated with cancer progression

**Fig. 4:**
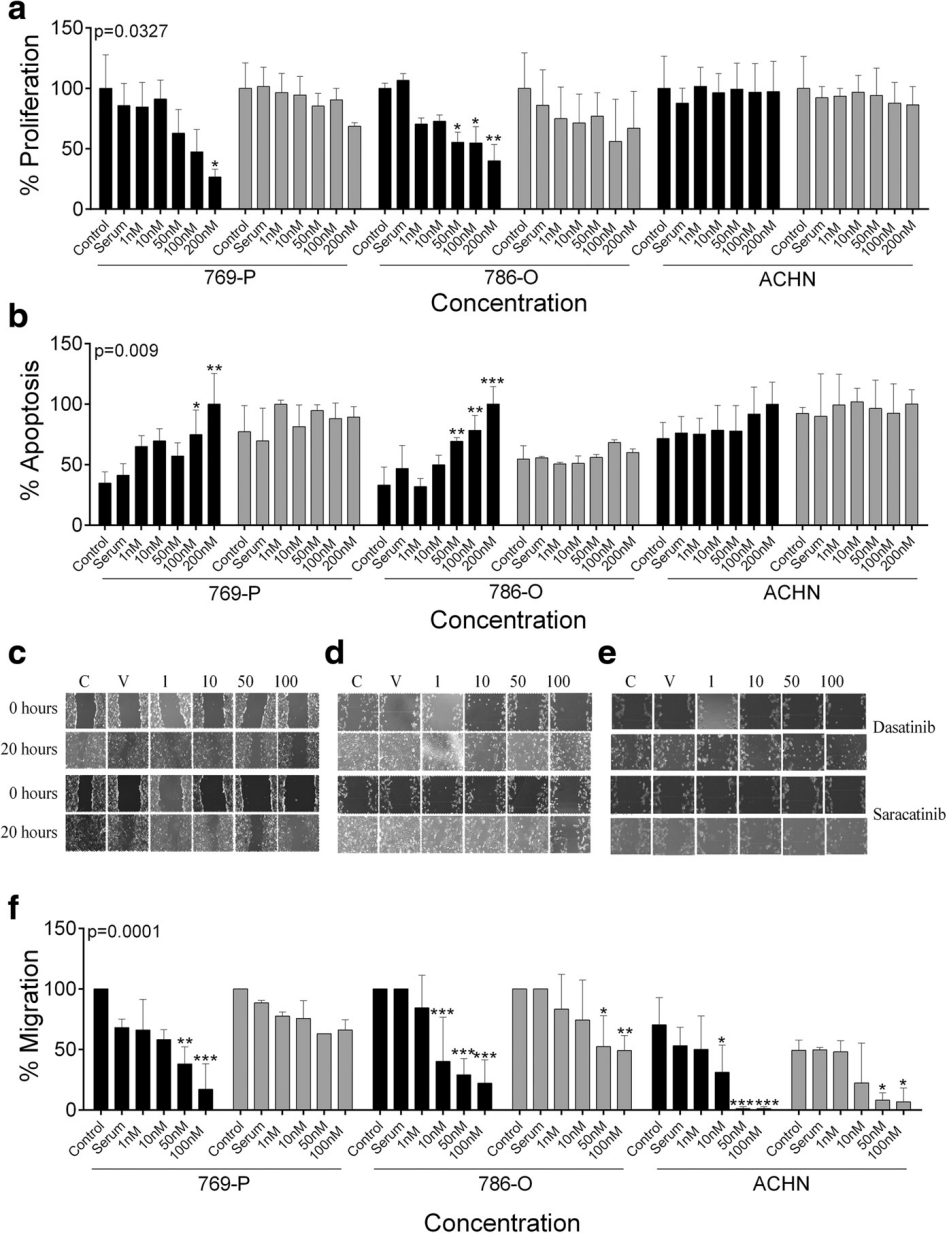
Dasatinib affects multiple oncogenic processes in non-metastatic ccRCC cell lines. **a**-**b** Graph showing the effect of 1, 10, 50 and 100 nM dasatinib (black bars) or saracatinib (grey bars) on (**a**) proliferation and (**b**) apoptosis in non-metastatic, 769-P and 786-O and metastatic ACHN cells. **c**-**e** Photographs and (**f**) quantification graphs showing the effect of the inhibitors on migration as measured via wound healing in the three cell lines (*n* = 3; * = 0.05, ** = 0.01, *** = 0.001)

Dasatinib showed a significant dose dependent response on apoptosis, proliferation and motility in early non-metastatic 769-P and 786-O ccRCC cell lines (Fig. 4). In contrast in saracatinib treated cells, there was no statistically significant response observed for any of these functional outputs; however, there was a trend towards inhibition of wound healing (Fig. 4c, d, f). In metastatic ACHN cells, only wound healing was significantly affected by dasatinib and saracatinib (Fig. 4e, f).

### SFK inhibitors do not act via the prototypical SFK, Src kinase, in early RCC cells

**Fig. 5:**
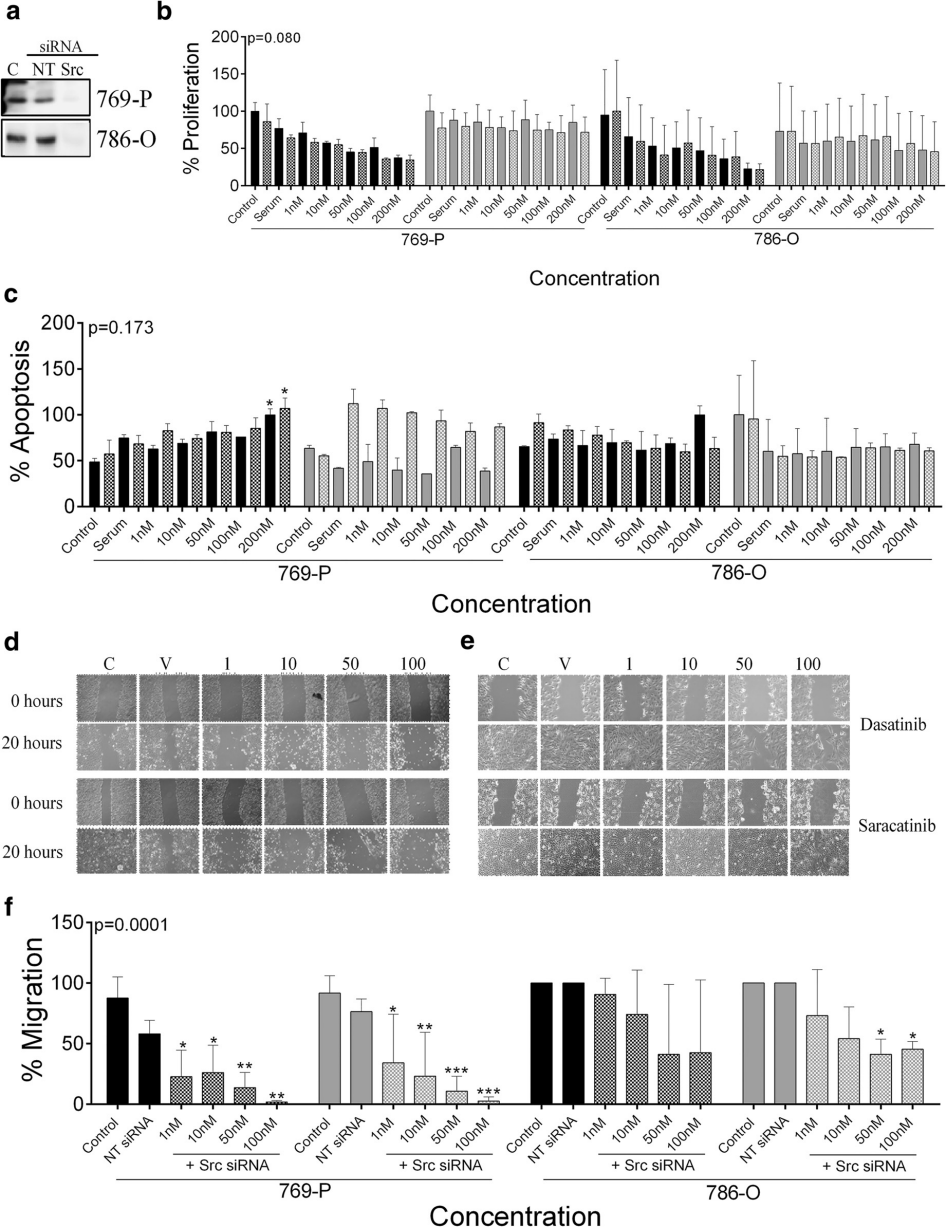
Dasatinib does not function via Src kinase in non-metastatic ccRCC cell lines. **a** Western blots and showing siRNA knockdown of NT or Src kinase in 769-P and 786–O. **b**-**c** Graph showing effect of 1, 10, 50 and 100 nM Dasatinib (dark bars) and saracatinib (light bars) on (**b**) proliferation and (**c**) apoptosis in the presence of NT (solid bars) and Src siRNA (checked bars) in 769-P and 786-O cells. **d**, **e** Photographs and (**f**) quantification graphs showing effects of inhibitors on migration as assessed by wound healing in 769-P and 786-O cells (*n* = 3-4; * = 0.05, ** = 0.01, *** = 0.001)

In early non-metastatic 769-P and 786-O ccRCC cells, src kinase was silenced using SMARTpool siRNA, with a knockdown of approximately 85 % for both cell lines (Fig. 5a). In silenced 769-P and 786-O cells, dasatinib significantly decreased proliferation to a similar extent to that seen in the non-silenced cells (Fig. 5b). Similarly, dasatinib still increased apoptosis and inhibited wound healing in 769-P silenced cells, as did saracatinib (Fig. 5c-f).

## Discussion

In the current study, the role of SFK in ccRCC were investigated. High nuclear expression of Lyn and downstream paxillin was associated with poor prognosis in our patient cohort and cell line studies demonstrated that dasatinib could induce apoptosis and inhibit proliferation. Nuclear accumulations of Lyn and paxillin have previously been observed in solid tumours and are both associated with inducing cell proliferation [18]. Lyn was reported to increase cell proliferation in breast cancer cell lines [18], via binding to and phosphorylating epidermal growth factor receptor (EGFR) in the cytoplasm and chaperoning EGFR translocation to the nucleus, whereby Lyn acted as a transcription factor in the up-regulation of expression of Cyclin D1, inducible nitric oxide synthase (iNOS) and other pro-oncogenic factors [18]. Both the nuclear accumulation of Lyn and induction of proliferation could be blocked by dasatinib [19]. Therefore, we hypothesis that a similar process may occur in ccRCC as nuclear Lyn is associated with poor prognosis in our clinical specimens and dasatinib induces apoptosis and inhibits cell proliferation and migration in ccRCC cell lines independent of Src kinase.

Nuclear paxillin has also been observed to increase cell proliferation via transcription of Cyclin D1 in prostate cancer cells, by acting as a nuclear scaffold, to promote extracellular-regulated kinase (ERK) phosphorylation of the ETS domain-containing protein (Elk-1) [20]. Paxillin may therefore be acting as a scaffold within a SFK protein complex in ccRCC; however this requires further investigation. Overall, these results suggest, inhibition of Lyn may have therapeutic potential in ccRCC, but further mechanistic studies are required to confirm these findings. Furthermore, dasatinib may not be the appropriate inhibitor to employ clinically due to the associations we observed with good prognosis and other family members. Development of inhibitors selective for Lyn over other family members may be more clinically relevant.

In addition to the poor prognosis observed in this study with nuclear Lyn and paxillin, good prognosis was also observed with membrane expression of Fyn, Hck, Yes and activated FAK, stressing the importance of analysing different cellular localization for SFKs in ccRCC. As Fyn and Yes are known to associated with FAK at the cell membrane, it was reassuring that all three were associated with the same prognosis; however it has previously been reported that their role was to regulate cell adhesion, migration, and invasion in response to integrins, which would lead to poor prognosis [18]. In the current study we have observed that Fyn, Hck, Yes and activated FAK are all associated with good prognosis and to date their role as potential tumour suppressors within this complex has not been widely investigated, and reveals a potential new role for these SFKs in ccRCC.

The current study did not observe any significant association between cytoplasmic localisation of any SFK or activated FAK and DSS. This is in contrast to previous work within our laboratory which showed that cytoplasmic FAK (Y^861^) was significantly associated with decreased DSS. This difference may be a result of the differing cohort sizes as the initial study only included 57 patient whereas this study includes 192, therefore the association seen in the smaller cohort may have been diluted out as the cohort size has increased.

The current study observed that dasatinib affects activation of SFKs via phosphorylation at Y^419^ to increase apoptosis and decrease proliferation but not saracatinib. Dasatinib may achieve this by blocking the Src homology 2 (SH2) domain of its target SFK inhibiting binding of auto-phosphorylated FAK (Y^397^), which is required for SFK activation via phosphorylation at Y^419^. Therefore, the observed increase in FAK phosphorylation at Y^397^ may be a compensatory mechanism to swamp the SFK SH2 domain with FAK (Y^397^) to overcome dasatinib inhibition. The data also suggests that saracatinib acts at a different site within the protein that does not interfere with the SH2 domain binding of FAK or the Y^419^ phosphorylation site. Both inhibitors were observed to be active in the early 786-O and 769-P cell lines and not the metastatic ACHN cells. This is most likely due to our groups previously reported observation that SFKs are highly expressed in T2 stage tumours to stimulate cell migration and invasion (similar to the 786-O and 769-P cells). However, once the cells have metastasised levels of SFKs are down-regulated to nominal levels (equivalent to the ACHN cells) [12]. Therefore, although SFKs are present in ACHN cells they may not be active. Further investigation is required to confirm this hypothesis.

## Conclusions

In conclusion, cellular localization of SFKs in ccRCC tumours appears to be a significant factor influencing their role as a tumour suppressor or enhancer. Lyn and paxillin may be potential biomarkers to select the patients most likely to benefit from treatment with SFK inhibitors such as dasatinib. However, development of inhibitors specific for different SFKs could be an attractive novel therapeutic approach for ccRCC patients.

### Availability of data and materials

The dataset supporting the conclusions of this article is available in the Greater Glasgow and Clyde Biorepository and Safe haven, https://www.nhsgbr.org.uk (JE-Renal-TMA).

## Abbreviations

ccRCC: clear cell renal cancer
VEGF TKi: vascular endothelial growth factor receptor tyrosine kinase inhibitor
mTOR: mammalian target of rapamycin
SFK: Src family kinase
siRNA: small interfering ribonucleic acid
NT: non-targeting
TMA: tissue microarray
FAK: focal adhesion kinase
EDTA: ethylenediaminetetraacetic acid
DAB: chromagen 3,3-diaminobenzidine
EMEM: essential modified Eagle’s medium
HRP: horseradish peroxidase
WST: water soluble tetrazolium salt
ABTS: 2,2-azinobis (3-ethylbenzothiazoline-6-sulphonic acid)
DSS: disease specific survival
EGFR: epidermal growth factor receptor
iNOS: inducible nitric oxide synthase
ERK: extracellular-regulated kinase
Elk-1: ETS domain-containing protein
SH2: Src homology 2
